# Elevated plasma thrombomodulin and angiopoietin-2 predict the development of acute kidney injury in patients with acute myocardial infarction

**DOI:** 10.1186/cc13876

**Published:** 2014-05-16

**Authors:** Kuan-Liang Liu, Kuang-Tso Lee, Chih-Hsiang Chang, Yung-Chang Chen, Shu-Min Lin, Pao-Hsien Chu

**Affiliations:** 1 Division of Cardiology, Department of Internal Medicine, Chang Gung Memorial Hospital, Chang Gung University, College of Medicine, 199 Tun-Hwa North Road, Taipei 105, Taiwan; 2 Department of Nephrology, Chang Gung Memorial Hospital, Chang Gung University, College of Medicine, 199 Tun-Hwa North Road, Taipei 105, Taiwan; 3 Department of Thoracic Medicine, Chang Gung Memorial Hospital, Chang Gung University, College of Medicine, 199 Tun-Hwa North Road, Taipei 105, Taiwan; 4 Healthcare Center, Chang Gung Memorial Hospital, Chang Gung University, College of Medicine, 199 Tun-Hwa North Road, Taipei 105, Taiwan; 5 Heart Failure Center, Chang Gung Memorial Hospital, Chang Gung University, College of Medicine, 199 Tun-Hwa North Road, Taipei 105, Taiwan

## Abstract

**Introduction:**

Acute kidney injury (AKI) following acute myocardial infarction (AMI) is associated with unfavorable prognosis. Endothelial activation and injury were found to play a critical role in the development of both AKI and AMI. This pilot study aimed to determine whether the plasma markers of endothelial injury and activation could serve as independent predictors for AKI in patients with AMI.

**Methods:**

This prospective study was conducted from March 2010 to July 2012 and enrolled consecutive 132 patients with AMI receiving percutaneous coronary intervention (PCI). Plasma levels of thrombomodulin (TM), von Willebrand factor (vWF), angiopoietin (Ang)-1, Ang-2, Tie-2, and vascular endothelial growth factor (VEGF) were measured on day 1 of AMI. AKI was defined as elevation of serum creatinine of more than 0.3 mg/dL within 48 hours.

**Results:**

In total, 13 out of 132 (9.8%) patients with AMI developed AKI within 48 hours. Compared with patients without AKI, patients with AKI had increased plasma levels of Ang-2 (6338.28 ± 5862.77 versus 2412.03 ± 1256.58 pg/mL, *P* = 0.033) and sTM (7.6 ± 2.26 versus 5.34 ± 2.0 ng/mL, *P* < 0.001), and lower estimated glomerular filtration rate (eGFR) (46.5 ± 20.2 versus 92.5 ± 25.5 mL/min/1.73 m^2^, *P* < 0.001). Furthermore, the areas under the receiver operating curves demonstrated that plasma thrombomodulin (TM) and Ang-2 levels on day 1 of AMI had modest discriminative powers for predicting AKI development following AMI (0.796, *P* <0.001; 0.833, *P* <0.001; respectively).

**Conclusions:**

Endothelial activation, quantified by plasma levels of TM and Ang-2 may play an important role in development of AKI in patients with AMI.

## Introduction

Acute kidney injury (AKI) is a common complication of acute myocardial infarction (AMI). The incidence of AKI ranged from 9.6 to 19% in hemodynamically stable AMI patients [1-3] and up to more than 50% in AMI patients with cardiogenic shock [4]. Worsening renal function during AMI, even transient, is associated with adverse outcome and long-term mortality [2,5]. The mechanism of AKI in patients with AMI is multifactorial, including underlying renal insufficiency, hemodynamic alterations, changes in volume status, neurohormonal activation, and medical therapies such as diuretics and agents that may affect the renin–angiotensin–aldosterone axis, immune-mediated damages, and the use of contrast medium [6].

Recent evidence suggests that endothelial injury of the renal vasculature may play an important role in the pathogenesis of both early and chronic ischemic AKI [7]. Early alterations in peritubular capillary blood flow during reperfusion have been documented to associate with loss of normal endothelial cell function [7]. Several molecules, such as thrombomodulin (TM) and angiopoietins, were reported to be connected with endothelial injury [8,9]. TM is a membrane-bound glycoprotein predominantly located at the vascular endothelium [10]. Once bound to thrombin, TM accelerates protein C activation, triggers its own proteolytic degradation, and releases the resultant soluble form of thrombomodulin (sTM) to serum [11]. Endothelial injury has been reported to be the unique stimulation for sTM to be released from the surfaces of endothelial cells [12]. Increased levels of plasma sTM can therefore serve as a marker of endothelial injury, and have been found to be associated with worse outcomes in patients with acute coronary syndrome [13]. Furthermore, increased plasma levels of sTM have been reported to be associated with kidney injury induced by sepsis [14] and diabetes [15].

Angiopoietin (Ang)-1, Ang-2, and their endothelial cell-specific tyrosine kinase receptor, Tie-2, interact with vascular endothelial growth factor (VEGF) to mediate endothelial activation. Ang-1 exerts protective effects and limits activation of the endothelium by exogenous cytokine. Ang-1 is anti-inflammatory by inhibiting VEGF-induced blood-vessel formation and adhesion molecule expression. Ang-1 also downregulates VEGF expression and attenuates thrombin-induced permeability [16]. In contrast, Ang-2 triggers an inflammatory response by activating the endothelium and increasing its permeability [8]. With the important roles of angiopoietins in endothelial activation and vascular barrier breakdown being identified, applicability of these molecules to serve as biomarkers of critical illness was explored in several studies [17]. For example, plasma levels of Ang-2, Tie-2, and VEGF but not Ang-1 are found to be increased in patients with acute coronary syndrome [18]. Moreover, circulating Ang-2 was documented as a strong and independent predictor for mortality of patients with dialysis-dependent AKI in the ICU [19].

Although endothelial activation and injury have been suggested to be significant in AKI caused by sepsis or diabetes, what roles they may play in AKI development following an AMI event have not been elucidated. This pilot study aimed to determine whether the plasma markers of endothelial injury and activation could serve as independent predictors for AKI in patients with AMI.

## Materials and methods

### Study population

This prospective study was conducted from March 2010 to July 2012 and enrolled consecutive patients with AMI receiving percutaneous coronary intervention (PCI). The study was approved by the Institutional Review Board of Chang Gung Memorial Hospital, Taiwan (Institutional Review Board No. 99-0140B) and conformed to the tenets of the Declaration of Helsinki. All enrolled patients provided informed consent.

The inclusion criteria were patients with AMI receiving PCI. Exclusion criteria were patients with chronic kidney disease requiring dialysis or with previous kidney transplantation. Patients with preexisting renal impairment were included in the study.

AMI was diagnosed by the universal definition published in 2007 [20]. AKI was diagnosed according to the definition proposed by the AKI Network [21]; that is, an increase in the level of serum creatinine >0.3 mg/dl within 48 hours.

### Baseline and procedural data

The demographic data of patients were collected on the first day of AMI. The collected information included body mass index, blood pressure, heart rate, smoking status, and Killip class in addition to previous diagnosis of hypertension, hyperlipidemia, diabetes, and coronary artery disease. The left ventricular ejection fraction (LVEF) acquired by modified Simpson’s method with echocardiography was obtained on admission.

Patients’ serum troponin I and creatine kinase-MB levels were measured on admission and every 8 hours. The peak creatine kinase-MB level was used in the statistical analysis. The estimated glomerular filtration rate (eGFR) was calculated by the Modification of Diet in Renal Disease formula [22]. High-sensitive C-reactive protein (hsCRP), hemoglobin, platelet, and leukocyte counts were measured on admission.

The PCI procedure was performed according to contemporary interventional guidelines. Patients were pretreated with standard-dose unfractionated heparin, aspirin and clopidogrel, followed by the treatment including anti-platelet therapy, lipid-lowering and cardioprotective drugs according to current treatment guidelines [23]. The door-to-balloon time, use of the drug-eluting or bare metal stent, and the amount of contrast medium used in the index procedure were recorded. Number of vessels involved was defined with binary fashion; that is, the severity of ≥50% stenosis would be regarded as significant.

### Enzyme-linked immunosorbent assay

Plasma was collected from blood taken on admission. The concentrations of sTM, von Willebrand factor, Ang-1, Ang-2, Tie-2, and VEGF were determined by enzyme-linked immunosorbent assay (R&D system, Minneapolis, MN, USA) following the manufacturers’ instructions. The measured values were compared between patients with and without AKI.

### Statistical analysis

All data were expressed as mean ± standard deviation or percentage. As the distribution of most continuous variables was skewed, nonparametric analyses were performed. Comparison of continuous and nominal variables between two groups was performed with the Mann–Whitney test and the chi-square test, respectively. Receiver operating characteristic (ROC) curves for day 1 plasma levels of Ang-2 and sTM to predict the development of AKI were plotted and the respective areas under the curves were calculated. *P* < 0.05 using a two-sided test was considered statistically significant. All analyses were carried out using the SPSS software package, version 20.0 (SPSS, Inc., Chicago, IL, USA).

## Results

One hundred and thirty-two patients who were diagnosed with AMI from March 2010 to July 2012 were consecutively recruited for this study. Among them, 130 patients had acute ST-elevation myocardial infarction and two patients had non-ST-elevation myocardial infarction. Thirteen of 132 (9.8%) patients developed AKI within 48 hours. The demographic characteristics, biomarkers, clinical presentation, and infarction type are presented in Table 1. Compared with patients without AKI, patients with AKI had higher incidence of hypertension (84.6 vs. 44.5%, *P* = 0.008) and coronary artery disease history (53.8 vs. 10.9%, *P* < 0.001), increased peripheral blood leukocyte count (15,954 ± 8,647 vs. 11,160 ± 3,646/ml, *P* <0.001), plasma levels of hsCRP (44.86 ± 63.82 vs. 13.44 ± 28.38 mg/l, *P* = 0.001), Ang-2 (6,338.28 ± 5,862.77 vs. 2,412.03 ± 1,256.58 pg/ml, *P* = 0.033) and sTM (7.6 ± 2.26 vs. 5.34 ± 2.0 ng/ml, *P* < 0.001). In addition, patients with AKI had higher Killip class (61.5 vs. 20.2% ≥3, *P* = 0.001), lower eGFR (46.5 ± 20.2 vs. 92.5 ± 25.5 ml/minute/1.73 m^2^, *P* < 0.001), and less use of clopidogrel (84.6 vs. 99.2%, *P* =0.026). No significant differences were found for age, gender, diabetes mellitus, hyperlipidemia, smoking status, body mass index, blood pressure, heart rate, door-to-balloon time, amount of contrast medium, LVEF, hemoglobin, platelet count, hsCRP, creatine kinase-MB, VEGF, von Willebrand factor, Ang-1, Ang-2, number of vessels involved, type of stent used, aspirin, angiotensin-converting enzyme inhibitor/angiotensin receptor blocker, and statin between the two groups. Multivariate analysis was not performed due to the low incidence of AKI events in the study cohort.

**Table 1:** Demography, clinical presentation, biomarkers, and infarction characteristics of patients with acute myocardial infarction

	**All patients**	**With AKI**	**Without AKI**	***P *** **value**
	**(*n *** **= 132)**	**(*n *** **= 13)**	**(*n *** **= 119)**	
**Demography**				
Age (years)	59 ± 13	66 ± 14	59 ± 13	0.067
Male gender	118 (89.4%)	12 (92.3%)	106 (89.1%)	1.000
Hypertension	64 (48.5%)	11 (84.6%)	53 (44.5%)	0.008
Diabetes	36 (27.3%)	6 (46.2%)	30 (25.2%)	0.107
Hyperlipidemia	33 (25%)	3 (23.1%)	30 (25.2%)	1.000
Current smoker	84 (63.6%)	8 (61.5%)	76 (63.9%)	0.868
CAD history	20 (15.2%)	7 (53.8%)	13 (10.9%)	<0.001
Body mass index (kg/m^2^)	25.6 ± 3.7	25.7 ± 3.6	25.6 ± 3.7	0.974
**Clinical presentation**				
Door-to-balloon time (minutes)	74 ± 42	73 ± 11	74 ± 44	0.909
Systolic blood pressure (mmHg)	139 ± 39	136 ± 66	140 ± 36	0.852
Diastolic blood pressure (mmHg)	89 ± 25	87 ± 48	89 ± 21	0.883
Heart rate (beats/minute)	77 ± 21	89 ± 41	75 ± 18	0.270
Killip class ≥3	32 (24.2%)	8 (61.5%)	24 (20.2%)	0.001
Killip class				
1	87 (65.9%)	3 (23.1%)	84 (70.6%)	
2	13 (9.8%)	2 (15.4%)	11 (9.2%)	
3	10 (7.6%)	4 (30.8%)	6 (5.0%)	
4	22 (16.7%)	4 (30.8%)	18 (15.1%)	
LVEF (%)	55 ± 13	46 ± 21	56 ± 11	0.112
eGFR (ml/minute/1.73 m^2^)	88.0 ± 28.5	46.5 ± 20.2	92.5 ± 25.5	<0.001
Contrast medium (ml)	249 ± 61	254 ± 33	248 ± 63	0.846
**Hematology and biomarkers of myocardial necrosis and inflammation**				
Leukocyte count (/ml)	11,632 ± 4,569	15,954 ± 8,647	11,160 ± 3,646	<0.001
Hemoglobin (g/dl)	14.7 ± 1.8	14.1 ± 1.9	14.8 ± 1.8	0.195
Platelet count (1,000/ml)	226 ± 67	271 ± 139	222 ± 53	0.222
Peak creatine kinase-MB (units/l)	280 ± 254	327 ± 495	275 ± 219	0.734
hsCRP (mg/l)	15.7 ± 27.4	44.86 ± 63.82	13.14 ± 20.38	0.001
**Biomarkers of endothelial injury**				
VEGF (pg/ml)	258.03 ± 373.74	398.10 ± 346.13	241.92 ± 374.86	0.154
Tie-2 (ng/ml)	17.50 ± 8.89	19.02 ± 6.43	17.33 ± 9.13	0.519
vWF (MU)	716.36 ± 216.37	734.31 ± 273.64	714.40 ± 261.12	0.795
Thrombomodulin (ng/ml)	5.56 ± 2.1	7.6 ± 2.26	5.34 ± 2.0	<0.001
Angiopoietin-1 (pg/ml)	29,082.88 ± 20,898.78	36,070.09 ± 21,123.43	28,319.57 ± 20,821.49	0.228
Angiopoietin-2 (pg/ml)	2,798.71 ± 2,439.26	6,338.28 ± 5,862.77	2,412.03 ± 1,256.58	0.033
**Infarction type**				
Culprit vessel				
LMCA	1 (1.3%)	0	1 (0.9%)	1.000
LAD	69 (52.3%)	8 (61.5%)	61 (51.3%)	0.481
LCx	10 (7.6%)	0	10 (8.4%)	0.597
RCA	52 (39.4%)	5 (38.5%)	47 (39.5%)	0.942
Grafts	0	0	0	
Multivessel disease	63 (47.7%)	8 (51.5%)	55 (46.2%)	0.294
Number of involved vessels				
1	68 (51.5%)	5 (38.5%)	29 (24.4%)	
2	38 (28.8%)	4 (30.8%)	22 (18.5%)	
3	25 (18.9%)	2 (15.4%)	17 (14.3%)	
Stenting				
Use of DES	75 (56.8%)	5 (38.5%)	70 (58.8%)	0.159
Use of BMS	36 (27.3%)	3 (23.1%)	33 (27.7%)	1.000
**Medications**				
Aspirin	127 (96.2%)	12 (92.3%)	115 (96.6%)	0.410
Clopidogrel	129 (97.7%)	11 (84.6%)	118 (99.2%)	0.026
Beta-blocker	120 (90.9%)	10 (76.9%)	110 (92.4%)	0.098
ACEI/ARB	96 (72.7%)	11 (84.6%)	85 (71.4%)	0.513
Statin	122 (92.4%)	11 (84.6%)	111 (93.2%)	0.256

Data presented as mean ± standard deviation or *n* (%). ACEI/ARB, angiotensin-converting enzyme inhibitor/angiotensin receptor blocker; AKI, acute kidney injury; BMS, bare metal stent; CAD, coronary artery disease; DES, drug-eluting stent; eGFR, estimated glomerular filtration rate; hsCRP, high-sensitive C-reactive protein; LAD, left anterior descending coronary artery; LCx, left circumflex coronary artery; LMCA, left main coronary artery; LVEF, left ventricular ejection fraction; RCA, right coronary artery; VEGF, vascular endothelial growth factor; vWF, von Willebrand factor.

**Figure 1:**
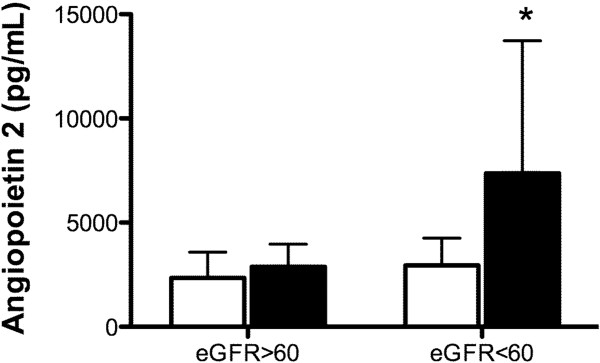
**Concentrations of angiopoietin-2 in patients with and without acute kidney injury.** Open bars, patients without acute kidney injury (AKI); solid bars, patients with AKI. *P < 0.05 compared with patients without AKI in the subgroup of estimated glomerular filtration rate (eGFR) < 60 ml/minute/1.73 m^2^. Data expressed as mean ± standard deviation.

**Figure 2:**
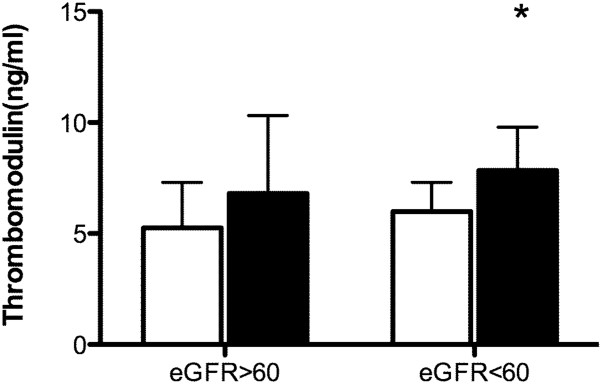
**Concentrations of thrombomodulin in patients with and without acute kidney injury.** Open bars, patients without acute kidney injury (AKI); solid bars, patients with AKI. *P < 0.05 compared with patients without AKI in the subgroup of estimated glomerular filtration rate (eGFR) < 60 ml/minute/1.73 m^2^. Data expressed as mean ± standard deviation.

In the study population, 109 patients had eGFR > 60 ml/minute/1.73 m^2^ while 24 patients had eGFR < 60 ml/minute/1.73 m^2^. The incidence of AKI in patients with eGFR < 60 ml/minute/1.73 m^2^ (10/24, 41.7%) was significantly increased compared with those with eGFR > 60 ml/minute/1.73 m^2^ (3/109, 2.8%; odds ratio, 37.18; 95% confidence interval, 6.61 to 111.7; *P* < 0.001). In patients with eGFR < 60 ml/minute/1.73 m^2^, plasma levels of Ang-2 (7,374 ± 6,357 pg/ml, *n* = 10 vs. 2,945 ± 1,314 pg/ml, *n* = 13; *P* = 0.023) and TM (7.84 ± 1.95 ng/ml, *n* = 10 vs. 5.99 ± 1.32 ng/ml, *n* = 13; *P* =0.013) were increased in patients with AKI compared with those without (Figure 1). In contrast, plasma levels of Ang-2 (2,886 ± 1,077 pg/ml, *n* = 3 vs. 2,347 ± 1,240 pg/ml, *n* = 106; *P* = 0.458) and TM (6.8 ± 3.52 ng/ml, *n* = 3 vs. 5.26 ± 2.05 ng/ml, *n* = 106; *P* =0.209) were similar in patients with and without AKI if their eGFR was > 60 ml/minute/1.73 m^2^ (Figure 2).

As shown in Table 2, the ROC curves of Ang-2 and sTM were plotted in predicting development of AKI 48 hours following AMI. Areas under the ROC curves showed that plasma levels of Ang-2 and TM on day 1 had modest discriminative powers in development of AKI (0.833; 95% confidence interval, 0.737 to 0.928; *P* <0.001; and 0.796; 95% confidence interval, 0.681 to 0.911; *P* <0.001, respectively). The cutoff values for Ang-2 and sTM in predicting AKI were 2,578.4 pg/ml and 5.44 ng/ml, respectively.

**Table 2:** Acquisition of the areas under the receiver operating characteristic curves for day 1 plasma levels of angiopoietin-2 and thrombomodulin to predict development of acute kidney injury within 48 hours after acute myocardial infarction

	**AUROC**	**95% CI**	***P *** **value**	**Cutoff value**	**Sensitivity**	**Specificity**
Angiopoietin-2	0.833	0.737 to 0.928	<0.001	2,578.4 pg/ml	0.846	0.689
Thrombomodulin	0.796	0.681 to 0.911	<0.001	5.44 ng/ml	0.846	0.622

AUROC, area under the receiver operating characteristic curves; CI, confidence interval.

The plasma levels of sTM in patients with multivessel disease (5.706 ± 0.332 ng/ml) were significantly lower than those in patients without (6.488 ± 0.302 ng/ml, *P* = 0.047). Compared with patients with Killip class 1, those with Killip class 2 to 4 had significantly higher Ang-2 levels (3,969 ± 673.2 vs. 2,773 ± 254.2 pg/ml, *P* = 0.009). The plasma concentration of Ang-2 in patients with LVEF < 45% (3,786 ± 566.8 pg/ml) was significantly higher than that in patients with LVEF > 45% (2,856 ± 211.3 pg/ml, *P* = 0.021).

## Discussion

The study demonstrated that 13 of 132 (9.8%) patients developed AKI 48 hours after the event of AMI. Compared with patients without AKI, patients with AKI had higher leukocyte count, plasma levels of eGFR, hsCRP, sTM, and Ang-2. In patients with eGFR < 60 ml/minute/1.73 m^2^, plasma levels of Ang-2 and TM were increased in patients with AKI compared with those without. Patients with multivessel disease had higher levels of plasma sTM than those without. Patients with impaired left ventricular function had increased Ang-2 levels.

Previous studies have shown an increase of the peripheral leukocyte count and hsCRP levels in patients with AMI-associated AKI [24,25]. Consistently, our data demonstrated that these two inflammation-related markers were increased in AMI patients with AKI. This result is suggestive of the role(s) that inflammation may play in mediating the development of AKI in AMI patients. Increased C-reactive protein is associated with platelet and clotting system activation, and decreased renal blood flow [26]. Increased C-reactive protein levels in AMI patients were therefore associated with development of AKI. Furthermore, C-reactive protein can mediate enhanced expression of adhesion molecules and plasminogen activator inhibitor-1. This may cause endothelium dysfunction and subsequent a proinflammatory, prothrombotic, and vasoconstrictive state of the endothelial system [27]. A previous prospective randomized study has shown that leukodepletion reduced postcardiopulmonary bypass renal injury in patients who underwent coronary bypass surgery [28]. Taken together, the inflammatory response following AMI may play a significant role in developing AKI.

We also showed that plasma levels of sTM were increased in AMI patients with AKI development. ROC curves demonstrated that the day 1 sTM level had a modest discriminative power in predicting AKI development within 48 hours after AMI. Since sTM has been shown to be a specific marker of endothelial injury, our data indicated that endothelial injury may be an essential mediator of kidney injury following AMI [12]. Endothelial injury may be triggered by altered hemodynamics, hypoxia, and inflammatory response following AMI. The injured endothelial cells in turn exert enhanced procoagulatory properties and may be responsible for the formation of a large number of microthrombotic foci, leading to organ microcirculation failures and subsequent complete organ failure [29]. Increased sTM is related to the severity of coronary disease, stroke, and peripheral occlusive arterial disease [30], and possibly associates with recurrence in patients with cardiovascular disease [31]. A possible connection between asymptomatic vascular atherosclerosis and sTM has been established previously [32]. In the present study, we showed that patients with multivessel disease, who may have a higher degree of vascular atherosclerosis or endothelial injury [33], had a significantly higher level of sTM than those without. The Ang–Tie2 signaling pathway in endothelial cells was reported to enhance local leukocyte recruitment and promote endothelial injury. This pathway may participate in vascular activation, proinflammatory responses, and leukocyte recruitment through complex signaling transduction in endothelial cells [34]. Previous studies have demonstrated that plasma levels of Ang-2, Tie-2, and VEGF but not of Ang-1 were increased in patients with AMI [18]. Elevated levels of Ang-2 were documented as an effective biomarker for AMI. Emerging evidence has indicated that Ang-2 is involved in critical illness-induced organ dysfunction [17]. Data in mice studies also showed that overexpression of Ang-2 in podocytes causes proteinuria, apoptosis of glomerular endothelium, and kidney injury [35]. As far as we can find from the literature, our data are the first to report that Ang-2 can serve as an independent clinical predictor of the development of AKI in patients with AMI. Furthermore, we demonstrated that poor cardiac function assessed by Killip class and echocardiography was related to the increased plasma level of Ang-2. This observation is consistent with a previous report showing that plasma levels of Ang-2 are progressively increased in patients with functional and hemodynamic decline of the heart [36]. Activation of endothelial cells, as indicated by plasma Ang-2 levels, may therefore be involved in development of AKI and heart failure in patients with AMI.

The plasma sTM and Ang-2 levels were increased in AMI patients with AKI. The corresponding areas under the ROC curve showed that both plasma sTM and Ang-2 levels on day 1 of AMI had modest discriminative power to predict AKI 48 hours after AMI. If further confirmed by large-scale prospective studies, these findings have great potential clinically. Patients may have sTM and Ang-2 measured early after AMI. If the levels are high, efforts to identify and treat the modifiable factors associated with kidney injury should be prompted. Patients may thus have better clinical outcome when the potential AKI is identified earlier than its onset and prevented beforehand. Further studies are warranted to evaluate the applicability of monitoring plasma sTM and Ang-2 levels in the management of AMI patients.

There are limitations of this study. First, the study is limited by the small sample size and single-center design. In addition, only a selective subgroup of AMI patients who received primary PCI was recruited. Patients who were not candidates for primary PCI due to severe comorbidities, unstable hemodynamic parameters, or delay diagnosis of AMI were excluded. Whether these potential markers could provide similar predictive capability to other subpopulations of patients with AMI remains to be investigated.

In summary, AMI patients with AKI had increased leukocyte count, plasma levels of hsCRP, sTM, and Ang-2 but decreased eGFR compared with those without AKI. The measurements taken on day 1 of AMI have modest discriminative power for predicting AKI 48 hours after AMI. Our data suggest that endothelial activation and injury play a significant role in mediating kidney injury in patients with AMI.

## Conclusion

AKI following AMI results in unfavorable prognosis, being related to both endothelial activation and injury. Our study demonstrates that the plasma markers, TM and Ang-2 levels on the first day of AMI have modest discriminative powers for prediction, and could serve as independent predictors for AKI in patients with AMI.

## Key messages

•AKI is a common complication of AMI.

•Endothelial activation and injury were found to play a critical role in the development of both AKI and AMI.

•Endothelial activation, quantified by plasma levels of TM and Ang-2, may play an important role in the development of AKI in patients with AMI.

## Abbreviations

AKI: acute kidney injury; AMI: acute myocardial infarction; Ang: angiopoietin; eGFR: estimated glomerular filtration rate; hsCRP: high-sensitive C-reactive protein; LVEF: left ventricular ejection fraction; PCI: percutaneous coronary intervention; ROC: receiver operating characteristic; sTM: soluble form of thrombomodulin; TM: thrombomodulin; VEGF: vascular endothelial growth factor.

## Competing interests

The authors declare that they have no competing interests.

## Authors’ contributions

K-LL and K-TL equally designed the study and prepared all of the data, and were responsible for the acquisition of data, analysis and writing the draft. C-HC prepared all of the data, and was responsible for the acquisition of data, analysis and writing the statistical part of the draft. Y-CC participated in the data collection, analysis and interpretation of data and revision. S-ML and P-HC equally initiated the study and supervised the acquisition of data, helped the final approval of the version to be published, and wrapped up the manuscript. All authors read and approved the final manuscript.
